# Why choose articaine over lidocaine for the removal of third molars? Systematic review and meta-analysis

**DOI:** 10.4317/jced.60682

**Published:** 2023-11-01

**Authors:** Emerson-Filipe-de Carvalh Nogueira, Renata-de Albuquerque-Cavalcanti Almeida, Bruno-Luiz-Menezes de Souza, Priscila-Lins Aguiar, Ricardo-José-de Holanda Vasconcellos

**Affiliations:** 1 MSc. Department of Oral and Maxillofacial Surgery, University of Pernambuco, Recife, PE, Brazil; 2 PhD. Professor of the Doctorate and Master in Oral and Maxillofacial Surgery. Department of Oral and Maxillofacial Surgery, University of Pernambuco, Recife, PE, Brazil; 3 MSc. Department of Oral and Maxillofacial Surgery, Hospital Regional do Agreste, Caruaru, PE, Brazil; 4 DDs. Department of Oral and Maxillofacial Surgery, University of Pernambuco, Recife, PE, Brazil; 5 PhD. Professor of the Doctorate and Master in Oral and Maxillofacial Surgery. Department of Oral and Maxillofacial Surgery, University of Pernambuco, Recife, PE, Brazil

## Abstract

**Background:**

The aim of the present study was to seek scientific evidence through a systematic review and meta-analysis for the choice of articaine over lidocaine in the removal of third molars.

**Material and Methods:**

Searches were performed of the MEDLINE/PubMed, EMBASE, Cochrane Library (CENTRAL), Web of Science, and SCOPUS databases as well as the grey literature.

**Results:**

Four hundred three articles were found, only 14 of which met the eligibility criteria. A total of 1114 third molars were removed: 557 with articaine and 557 with lidocaine. Articaine had a higher success rate than lidocaine (RR = 1.09, 95% CI: 1.03 to 1.15; *P*< 0.05), shorter subjective latency time (MD = -15.10, 95% CI: -21.57 to -8.63; *P*< 0.05), less intraoperative pain (MD = -6; *P*< 0.05), longer duration (MD = 68.86; *P*< 0.05), and less postoperative pain (MD = -3.05; *P*< 0.05).

**Conclusions:**

Based on the findings, articaine is superior to lidocaine for use in lower third molar surgeries due to the higher success rate, shorter time until the onset of action, greater control of intraoperative and postoperative pain, and longer duration of the anesthetic effect.

** Key words:**Articaine, lidocaine, third molar, impacted teeth.

## Introduction

The adequate use of local anesthetics during dental procedures is of the utmost importance in clinical practice. The effective control of pain, especially during surgeries, is an important factor in strengthening the trust between the patient and dentist (1).

The extraction of third molars is the most routinely performed surgical procedure by oral-maxillofacial surgeons but is often complex and challenging. The blocking of the inferior alveolar nerve is the most widely employed mandibular block technique in dentistry and also has the highest number of anesthetic failures, often with the need for reapplication (2).

The search for more effective, longer lasting anesthetics with a shorter onset is the object of discussion in the literature. Lidocaine has been widely used in clinical practice since it was first commercially available in 1948 and still occupies the position of “gold standard” anesthetic when its properties are compared to those of other substances. However, based on chemical differences and pharmacological properties, numerous studies have reported the superior efficacy of articaine in comparison to lidocaine (3,4). The main advantages are greater liposolubility and anesthetic potency (3,4,6,7), faster onset (8,9), greater duration of the anesthetic effect (10,11), and excellent diffusion in bone tissue (7,12,13).

Therefore, the aim of the present study was to seek scientific evidence through a systematic review with meta-analysis of the efficacy of articaine in comparison to lidocaine for use in third molar surgeries and analyze the side effects to assist in the choice of an anesthetic based on its risks and benefits.

## Material and Methods

This study was conducted following the Preferred Reporting Items for Systematic Reviews and Meta-Analyses (PRISMA statement) (14). The protocol for this study was submitted to PROSPERO and the study is registered under CRD42020204815.

-Guiding question 

The guiding question was based on the PICOS method (Population: patients submitted to the removal of lower third molars; Intervention: articaine; Comparation: lidocaine; Outcome: efficacy; Studies: randomized clinical trials): What are the reasons for the preference of articaine over lidocaine in the removal of lower third molars?

-Eligibility criteria

Randomized clinical trials with the use of 4% articaine and a vasoconstrictor (epinephrin) in comparison to lidocaine and a vasoconstrictor (epinephrin) for the blocking of the inferior alveolar nerve during the extraction of impacted, semi-impacted, or erupted lower third molars were included. No restrictions were imposed regarding the year of publication, sex of the patients, or language.

Observational studies, case series, case reports, narrative reviews, editorials, letters to the editor, studies not involving inferior alveolar nerve block, those that did not describe the blocking technique used, those with no intraoperative evaluation, and articles published in an incomplete form or for which it was not possible to contact the author, if necessary, were excluded.

-Search strategy 

Searches were performed of the MEDLINE/PubMed, EMBASE, Cochrane Library (CENTRAL), Web of Science, and SCOPUS databases between July and August 2020. The grey literature was searched through access to Clinical Trials, Open Grey, Biblioteca Digital de Teses e Dissertações (BDTD [Digital Library of Theses and Dissertations]), and Registro Brasileiro de Ensaios Clínicos (ReBEC [Brazilian Clinical Trial Registry]). A hand search was performed in the three main journals of the field (International Journal of Oral and Maxillofacial Surgery, Journal of Oral and Maxillofacial Surgery, and Journal of Cranio-Maxillo-Facial Surgery) as well as the reference lists of the studies included in the present systematic review. An expert with several publications on the topic was also consulted for the analysis of the included and excluded articles.

Medical Subject Headings (MeSH terms) were used for the search strategy with controlled descriptors in MEDLINE. To make the search more sensitive, non-controlled vocabulary was included with the use of keywords. Emtree terms were used for the search strategy in EMBASE. The search strategy for MEDLINE/PubMed was adapted for the searches of the Web of Science, Scopus, and Cochrane (CENTRAL) databases.

The search was performed with a combination of MeSH terms and keywords for the population, intervention, and comparison. The following was the strategy adopted for the MEDLINE/Pubmed database.

(“Third molar” OR “Third molars” OR “Wisdom tooth” OR “Wisdom teeth” OR “Tooth included” OR “Teeth included” OR “Tooth impacted” OR “Teeth impacted”) AND (Carticain OR Articain OR Articaine OR “Carticaine Hydrochloride” OR “Hydrochloride, Carticaine” OR Hoe-40045 OR “Hoe 40045” OR Hoe40045 OR Hoe-045 OR Hoe 045 OR Hoe045 OR Ultracaine) AND (2-(Diethylamino)-N-(2,6-Dimethylphenyl)Acetamide OR 2-2EtN-2MePhAcN OR Lignocaine OR “Lidocaine Carbonate” OR “Lidocaine Hydrocarbonate” OR “Lidocaine Hydrochloride” OR “Lidocaine Monohydrochloride” OR “Lidocaine Monoacetate” OR Xyloneural OR “Lidocaine Sulfate (1:1)” OR Octocaine OR Xylesthesin OR Xylocaine OR Xylocitin OR Dalcaine OR “Lidocaine Monohydrochloride, Monohydrate”).

-Outcomes

The primary outcome of the study was the success rate, which was defined as the execution of the procedure without the need for reapplication of the anesthetic after the beginning of surgery. The other outcomes considered were 1) subjective latency time (onset of anesthesia) measured in seconds (time between application of anesthesia and onset of numbness reported by the patient), 2) objective latency time measured in minutes (related to the absence of symptoms using the “prick test” on the anterior vestibular mucosa), 3) intraoperative pain defined by the patient and measured using the visual analog scale (0-100 mm), 4) duration of anesthesia measured in minutes and reported by the patient, and 5) side effects.

-Selection of articles

Two independent reviewers (specialists with master’s degrees in oral-maxillofacial surgery and traumatology [E.F.C.N. and B.L.M.S.]) performed the searches and article selection process. Cases of disagreement between the reviewers were resolved by a third reviewer (R.A.C.A.).

The article selection process was conducted in two steps. First, the titles and abstracts of the articles were read by the two main reviewers individually and independently for the preselection of articles with potential to answer the guiding question based on the inclusion and exclusion criteria. The first consensus meeting was then held to determine the level of inter-examiner agreement using the Kappa statistic. The two reviewers sought to come to a consensus on the selection of the articles at this time and cases of disagreement were resolved by a third reviewer. This step was performed for the preselection of articles to be submitted to full-text analysis.

The second step consisted of the reading of the complete texts of the preselected studies to determine which would be included in the systematic review. This step was also guided by the eligibility criteria and was followed by a consensus meeting in which a third reviewer made the decision in cases of a divergence of opinion between the two main reviewers.

-Data extraction

The two reviewers performed the extraction of the data using a Table developed specifically for the present review. The following information was recorded: type of study, mean age of study population, type of evaluation performed, anesthetic used in intervention and control groups with information on concentrations, sample size, success rate, time until onset of anesthesia (objective), time until onset of anesthesia (subjective), intraoperative pain, duration of anesthesia, postoperative pain, and side effects.

-Methodological appraisal of quality and risk of bias

The risk of bias of the studies included in the present review was appraised independently by two researchers (E.F.C.N and B.L.M.S.) using the revised Cochrane tool for risk of bias in randomized trials (RoB 2) (15) with the aid of the program available on the site riskofbias.info. The RoB 2 has five domains: randomization process, deviation from intended interventions, missing outcome data, measurement of the outcome, and selective reporting of the results. The scale furnishes a score for each domain and a global assessment of the risk of bias of each study, which was categorized as “low risk”, “high risk” or “with some concerns”.

-Synthesis of data

Meta-analysis was performed for the quantitative evaluation of the data extracted for the outcomes using the Review Manager software (RevMan version 5.3). Weighted mean differences (MD) and respective 95% confidence intervals (CI) were used to analyze the continuous variables. A *p-value* < 0.05 was considered indicative of statistical significance. Relative risk (RR) was estimated for dichotomous variables (success and side effects), whereas the MD was estimated for continuous variables (latency time, pain, and duration of anesthetic).

Heterogeneity among the was calculated using Cochran’s test and Higgins inconsistency test (I2). For Cochran’s test, studies with a *p-value* > 0.10 were considered indicative of low heterogeneity. For the Higgins test, values ≥ 50% indicate moderate heterogeneity and those above 75% indicate high heterogeneity. A random effects model was used in the occurrence of high heterogeneity.

## Results

The search strategy led to the retrieval of 411 papers: 68 in Embase, 123 in Cochrane, 110 in MEDLINE, 100 in Web of Science, two in Scopus, and eight in the grey literature. EndNote was used as the reference manager and identified 183 duplicates, which were removed, resulting in 220 papers with which the selection process was initiated with the aid of the same program.

**Figure 1:**
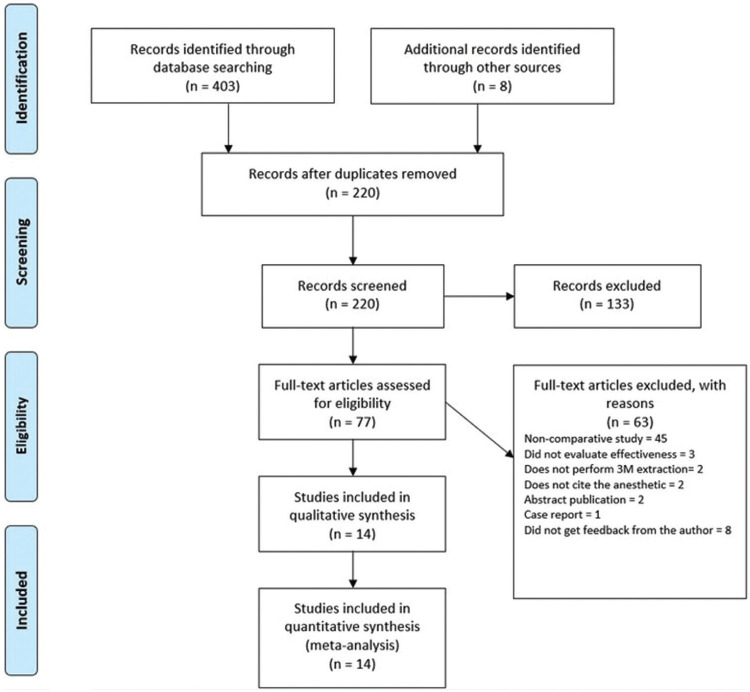
Flowchart of the research process.

After the reading of the titles and abstracts, 87 papers were selected for full-text analysis, 14 of which met the eligibility criteria and composed the present systematic review. Figure 1 displays the flowchart of the article selection process. A Table was created for the 74 articles excluded and the reasons for exclusion after the full-text analysis (Table 1,  Table 1 cont.). The level of agreement between the two reviewers was calculated using the Kappa coefficient (k = 0.933 for the titles and abstracts and k = 0.91 for the complete texts), which demonstrated excellent agreement.

**Table 1:**
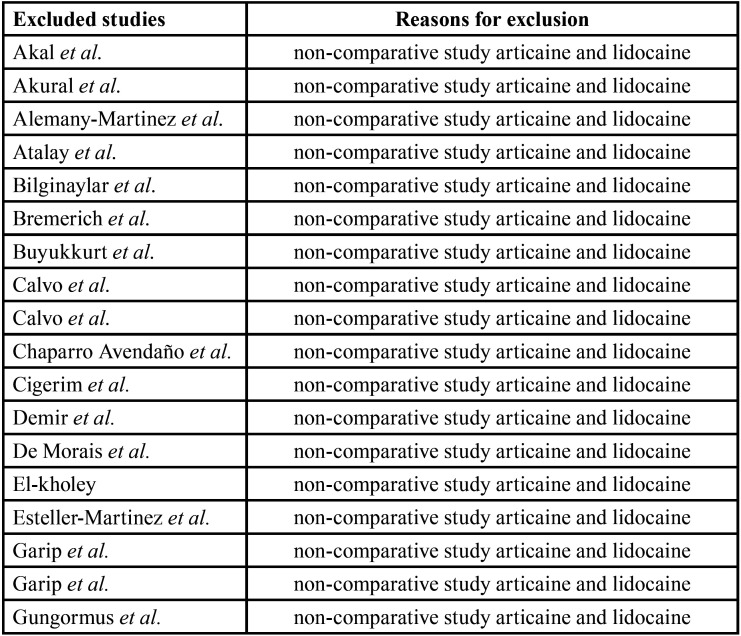
Excluded articles and reasons for their exclusions.

**Table 1 cont.**
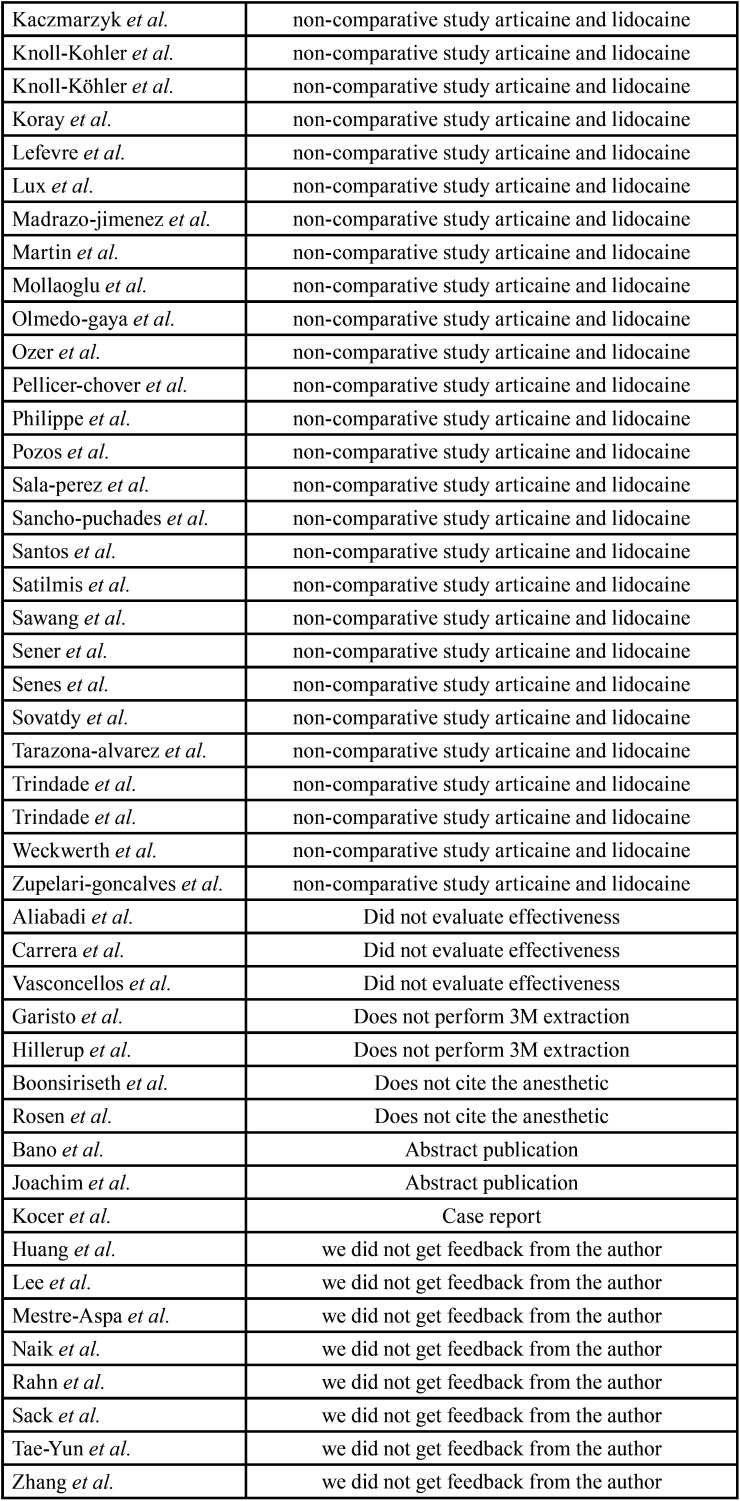
Excluded articles and reasons for their exclusions.

-Characteristics of studies 

Fourteen studies were included in the systematic review, all of which compared articaine to lidocaine. The majority (10 studies) (11,18-26) compared 4% with epinephrin 1:100,000 to 2% lidocaine with epinephrin 1:100,000. One study (10) compared 4% articaine with epinephrin 1:100,000 to 2% lidocaine with epinephrin 1:200,000. One study (27) compared 4% articaine with epinephrin 1:100,000 to 2% lidocaine with epinephrin 1:80,000. Another study (28) compared 4% articaine with epinephrin 1:200,000 to 2% lidocaine with epinephrin 1:200,000 and another study (29) compared 4% articaine with epinephrin 1:100,000 to 4% lidocaine with epinephrin 1:100,000.

A total of 1114 third molars were removed with local anesthesia and lower alveolar nerve block. Half (n = 557) involved the use of articaine (537 with 4% articaine and epinephrin 1:100,000 and 20 with 4% articaine and epinephrin 1:200,000) and the other half (n = 557) involved lidocaine (470 with 2% lidocaine and epinephrin 1:100,000, 30 with 2% lidocaine and epinephrin 1:200,000, 35 with 2% lidocaine and epinephrin 1:80,000, and 22 with 4% lidocaine and epinephrin 1:100,000).

All articles were randomized clinical trials. Seven used the split-mouth method (10,19,20,24,25,28,29) and seven used the parallel method (independent samples) (11,18,20,22,23,26,27). All articles investigated subjective latency of the onset of anesthesia, whereas only two investigated objective latency (20,29). In all articles, articaine had a faster onset than lidocaine.

Eight of the 14 studies assessed intraoperative pain (18-21,24,27-29). Articaine achieved better results for this outcome in all cases, except one article, which found a slight advantage with the use of lidocaine (24).

Thirteen studies assessed the duration of the anesthesia (10,11,17-23,25-28). Articaine achieved better results than lidocaine for this outcome in all cases.

Four studies (10,25,27,28) assessed postoperative pain and articaine achieved better results than lidocaine for this outcome in all cases.

Seven studies (18-20,24,27-29) assessed the success of anesthesia based on the non-need for reapplication. Articaine achieved better results for this outcome in all cases, except in one study (29), which found equal percentages for the two anesthetics. The mean success rate was 89.3% for articaine and 79.68% for lidocaine.

Side effects were investigated in nine studies (10,11,20,22-24,26,28,29), two of which (20,24) found nerve injury (four cases of temporary paresthesia) with the use of articaine, whereas no cases of paresthesia were cited with the use of lidocaine. One study (22) reported a case of tachycardia followed by syncope with the use of articaine and two studies (11,23) reported persistent trismus for at least one week – seven cases with the use of articaine and seven with the use of lidocaine.

The characteristics of the studies included in the present review are displayed in Table 2- Table 2 cont.-1.

**Table 2:**
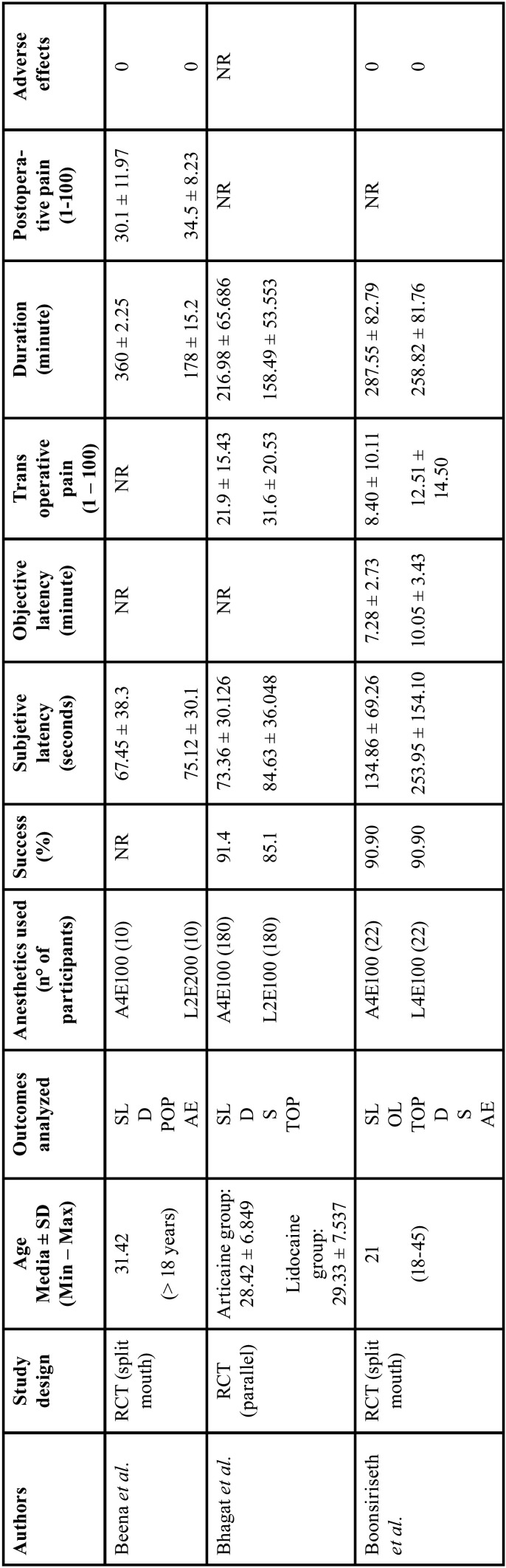
Characteristics of selected studies.

**Table 2 cont.**
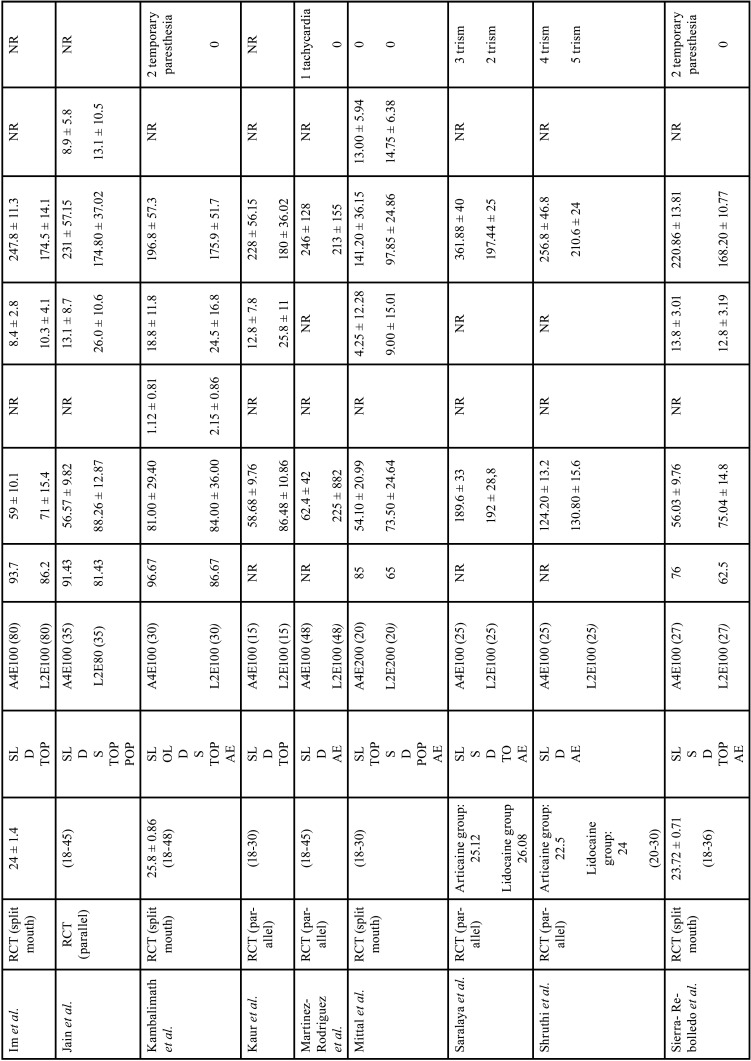
Characteristics of selected studies.

**Table 2 cont.-1:**
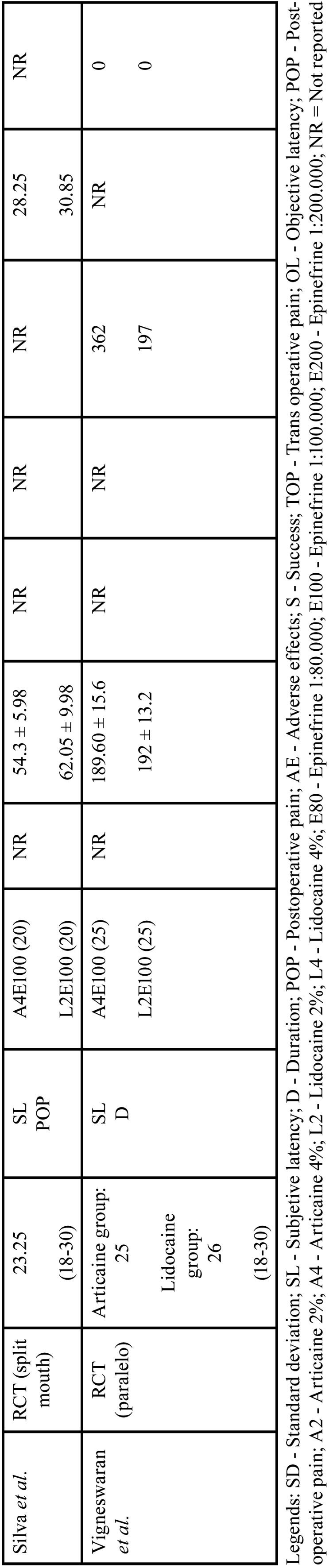
Characteristics of selected studies.

-Meta-analysis

Meta-analysis was performed with the aid of the RevMan 5.3 software for the following outcomes: success, latency time, intraoperative pain, duration of anesthesia, postoperative pain, and side effects.

**Figure 2:**
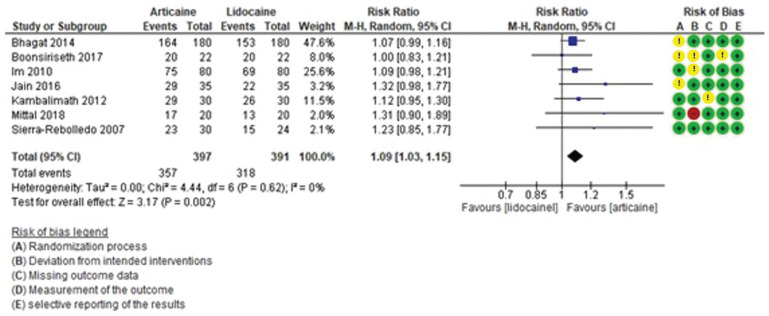
Forest plot of comparing success between articaine and lidocaine in lower third molar extraction.

Articaine achieved a higher success rate compared to lidocaine, as confirmed by the meta-analysis, which revealed RR = 1.09, 95% CI: [1.03, 1.15] with *P* = 0.002 and low heterogeneity (I² = 0%) (Fig. 2).

**Figure 3:**
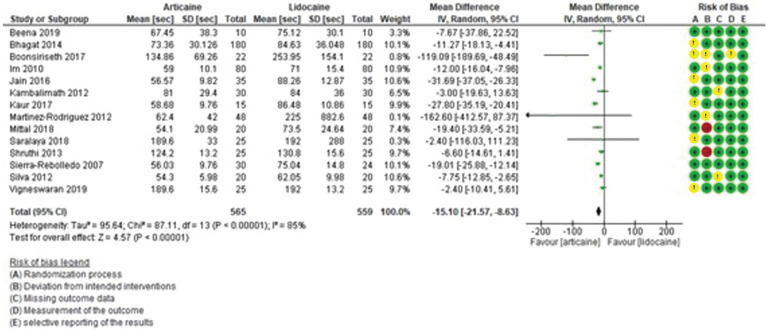
Forest plot of comparing latency subjetive between articaine and lidocaine in lower third molar extraction.

**Figure 4:**
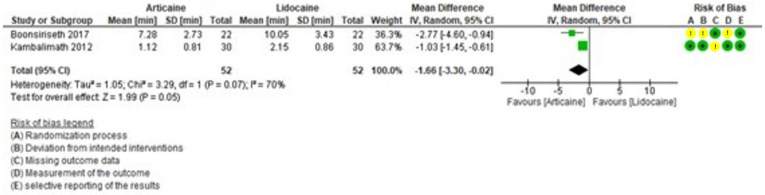
Forest plot of comparing latency objective between articaine and lidocaine in lower third molar extraction.

Subjective latency time was shorter with articaine than lidocaine. This result was statistically significant (MD = -15.10, 95% CI: [-21.57, -8.63]; *P* < 0.0001) and with high heterogeneity (I² = 85%) (Fig. 3). Objective latency was also shorter with articaine. The difference was not statistically significant (MD = -1.66, 95% CI: [-3.30, -0.02]; *P* = 0.05) (Fig. 4), but heterogeneity was moderate (I² = 70%).

**Figure 5:**
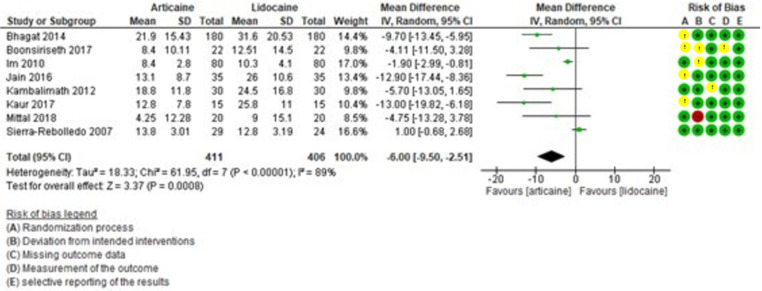
Forest plot of comparing intraoperative pain between articaine and lidocaine in lower third molar extraction.

The meta-analysis of intraoperative pain revealed high heterogeneity (I² = 89%) and a result favoring articaine over lidocaine (MD = -6.00, 95% CI: [-9.50, -2.51]; *P* = 0.0008) (Fig. 5).

**Figure 6:**
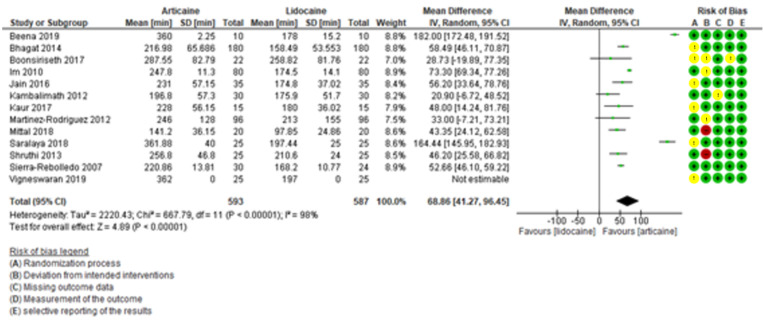
Forest plot of comparing duration between articaine and lidocaine in lower third molar extraction.

Figure 6 shows the results of the meta-analysis for duration of the anesthetic effect, which was longer with the use of articaine compared to lidocaine. The difference was statistically significant (MD = 68.86, 95% CI: [41.27, 96.45]; *P* < 0.00001) and heterogeneity was high (I² = 98%).

**Figure 7:**
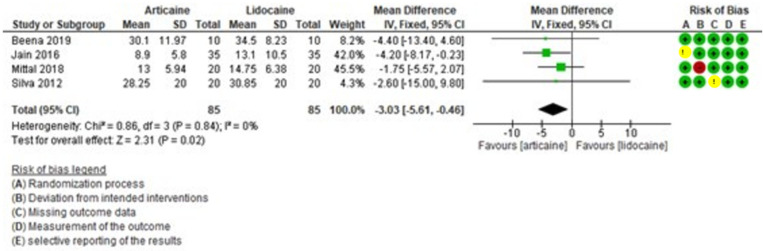
Forest plot of comparing postoperative pain between articaine and lidocaine in lower third molar extraction.

Figure 7 shows the results of the meta-analysis for postoperative pain, which also favored articaine over lidocaine. The difference was statistically significant (MD = -3.05, 95% CI: [-5.69, -0.42]; *P* = 0.02) and low heterogeneity was found among the studies (I² = 0%).

**Figure 8:**
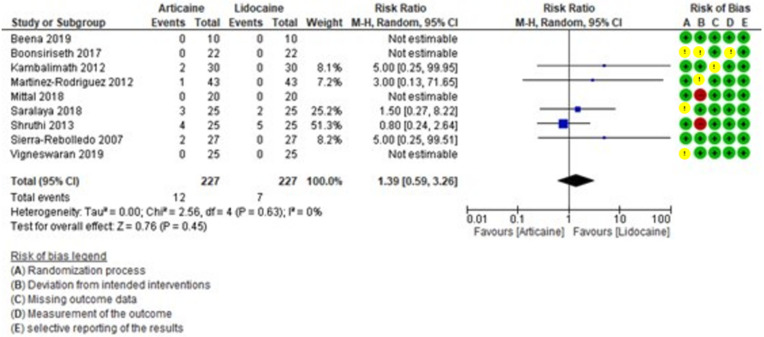
Forest plot of comparing adverse effects between articaine and lidocaine in lower third molar extraction.

The meta-analysis of side effects revealed low heterogeneity among the studies (I² = 0) and a 1.39-fold higher RR for articaine compared to lidocaine. However, the difference was not statistically significant (RR = 1.39, 95% CI: [0.59, 3.26]; *P* = 0.45) (Fig. 8).

-Appraisal of risk of bias

**Figure 9:**
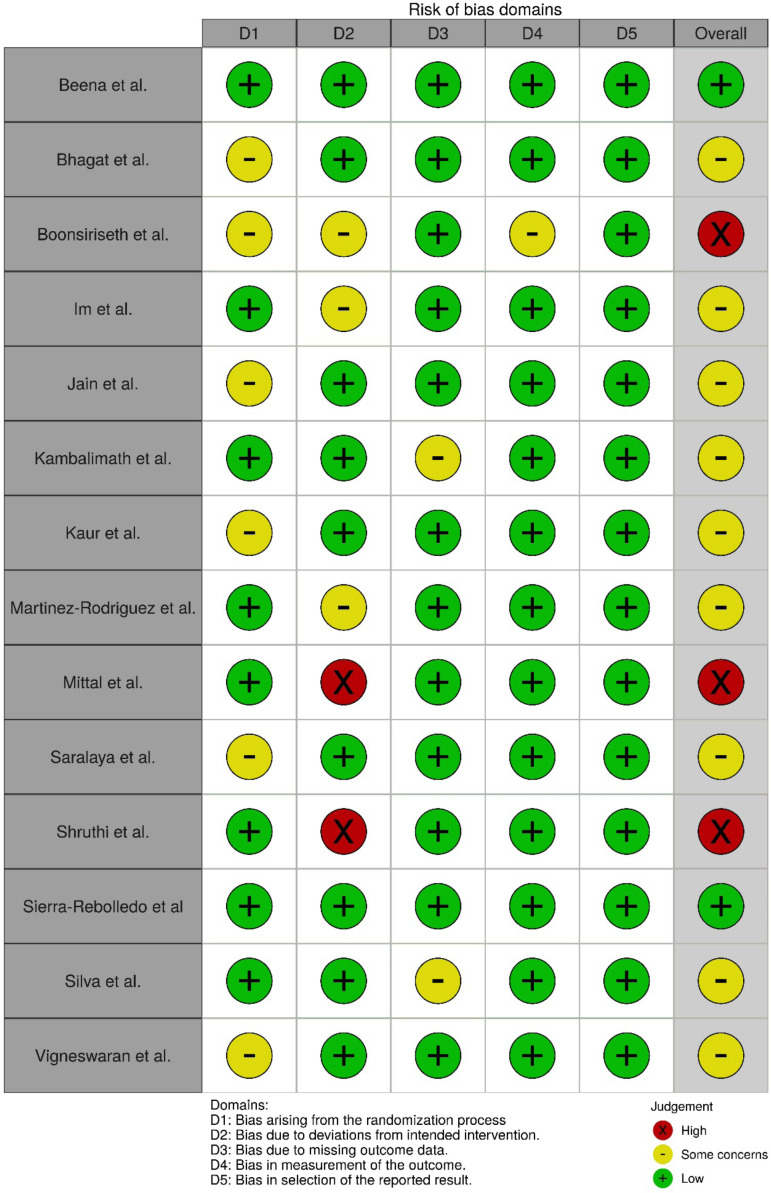
Bar plots graph for risk assessment of ROB2 bias (traffic light).

Figure 9 shows the results of the appraisal of the risk of bias using the “traffic light” of judgments on the domain level for each outcome.

The majority of studies (11,18-22,25-27) had some concerns regarding the risk of bias in the global appraisal. Only two studies (10,24) were classified as having a low risk of bias for the global appraisal using the RoB 2 criteria. Three studies (22,28,29) had a high risk of bias in the global appraisal. Two of these studies (23,28) had a high risk of bias regarding one of the criteria. Although the other study (29) did not have a high risk of bias for specific criterion, some concerns were found for three of the five criteria.

Regarding the randomization process, six studies (11,18,21,26,27,29) has some concerns. The randomization method was not specified and the studies failed to describe whether the allocation sequence was concealed until the participants were designated to the intervention.

For deviation from the intended interventions, three studies (19,22,29) were classified as having some concerns, as the studies suggested only performing blinding of the patients, but with no deviation of the intended intervention. In two studies (23,28), this criterion was classified as having a high risk of bias, as the authors either did not perform or did not describe the blinding of the study, suggesting that both the patients and health professionals in charge of the interventions were aware of the type of anesthetic employed in each intervention.

Only two studies (20,24) had some concerns regarding missing outcome data, as missing data beyond that established by the RoB 2 (availability of data should be higher than 95% for continuous outcomes) occurred in both studies. Moreover, the authors did not report the group to which the excluded sample belonged. All other studies analyzed (10,11,18,19,21-24,26-29) had a low risk of bias for this criterion.

Nearly all studies (10,11,18-28) had a low risk of bias regarding the measurement of the outcomes. Only one study (29) had some concerns for this criterion, as it is possible that the evaluator of the outcomes was aware of the intervention that the patients received, which could have influenced the evaluation of the results.

All studies (10,11,18-29) had a low risk of bias regarding the selective reporting of the results.

**Figure 10:**
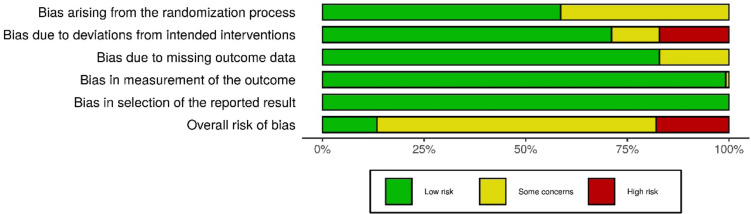
Bar plots graph for risk assessment of ROB2 bias (weighted bar plots).

Figure 10 shows the weighted findings of the distribution of risk of bias judgments in each domain taking the weight of the studies into consideration.

## Discussion

Lower third molar extraction is a common dental procedure, as these teeth can cause diseases in the oral cavity, such as pericoronitis, cysts, and tumors. During extraction, the both the surgeon and patient want the procedure to be comforTable and painless. Thus, the choice of a potent local anesthetic that provides good latency, sufficient duration, and few side effects assists in strengthening the dentist-patient relationship and enhances the success rate.

Lidocaine is considered a standard drug, as it was the first local amide anesthetic sold and is the most widely used in several countries (30) which is why it was chosen as the comparison group in the present investigation. Although lidocaine has proven to be a safe drug (31), its efficacy is inferior to other local anesthetics for the blocking of the inferior alveolar nerve during the removal of third molars (3,31).

Articaine emerged in clinical practice in Germany in 1976 and its use disseminated, entering North America in 1983 and the United Kingdom in 1998. It is currently indicated as a good anesthetic option for third molar removal due to its good diffusion in soft tissues and bone (7,12,13), rapid onset (3,5-7), and good potency (3,5-7). As disadvantages, some authors (32,33) state that articaine has been associated with an increase in the incidence of paresthesia and others (32,34) postulate that the 4% concentration of articaine, which is higher than that of other local anesthetics, is the reason for its neurotoxicity. However, these statements are contradicted by other studies (6,7,35-39).

As the removal of impacted lower third molars requires deep anesthesia of the pulp and soft tissues, this type of surgery is considered a good model for studying the efficacy of local anesthetics. Efficacy is generally evaluated using indirect variables, such as the need for additional injections (re-anesthesia) (27,40,41), total volume of the anesthetic solution applied (42,43), or the degree of intraoperative pain (20,27,44). To standardize the assessment instrument, the authors defined success as the non-need for re-anesthesia during surgery, which is the most widely used definition among studies, and found that the success rate was approximately 90% with articaine compared to 81% with the use of lidocaine.

Pain is one of the most commonly experienced symptoms in dentistry and nothing that a dentist can do for a patient is of greater importance than administering medication that prevents pain during dental treatment (18). The meta-analysis demonstrated the superiority of articaine over lidocaine regarding both intraoperative and postoperative pain. This is a clinically important result not only with regards to patient comfort, but also for the wellbeing of the surgeon by enabling a technically less stressful procedure. Only the study by Sierra-Rebolledo (24) reported greater intraoperative pain with the use of articaine. All other studies analyzed demonstrated the opposite (18-21,24,27-29). Only four studies assessed postoperative pain and all favored articaine over lidocaine (10,25,27,28), which is likely directly related to the duration of the anesthetic effect.

A recent systematic review (3), which was the only previous review published to compare the efficacy of articaine and lidocaine during the removal of lower third molars, also demonstrated the superiority of articaine. However, the study did not evaluate complications associated with the use of local anesthetics, which is an important outcome to consider when choosing these drugs. The study also imposed a restriction on the date of publication and presented errors in the extraction of the data. Therefore, the present systematic review was conducted to overcome these deficiencies and also included new clinical trials on the topic that have recently been published.

Local anesthetics with a rapid onset favor the surgical procedure. A longer time in the dental chair increases patient anxiety and contributes to the waste of active time by the dentist. A shorter latency time is an additional advantage of articaine. The meta-analysis of subjective latency time favored the use of articaine, although the mean difference was only 15.1 seconds, which does not provide evident clinical benefits. The objective latency time was also shorter with the use of articaine (1.66 minutes more with the use of lidocaine), but this difference did not achieve statistical significance. These findings differ from data reported by Zhang *et al*. (3).

Another clinical advantage of articaine is the duration of the anesthetic effect. This advantage was confirmed in the studies included in the present systematic review (10,11,18-24,26-29), with the effect of articaine greater than 60 minutes longer, on average, in comparison to lidocaine. This was confirmed in the meta-analysis, in which the mean difference was 68.86 minutes. Only one study (25) did not evaluate this outcome.

From the standpoint of safety, local anesthetics have been associated with some local and systemic side effects, such as dizziness, disorientation, seizures, tremors, and hemodynamic changes, including hypotension as well as respiratory and cardiovascular depression (43,44). These are rare events that can emerge as the result of an overdose or intravascular injection of a local anesthetic (44). De Morais *et al*. (44) conducted a clinical trial evaluating intraoperative vital signs with the use of 4% articaine and epinephrin 1:200,000 and found that the hemodynamic changes were not perceptible. In another study with a similar methodology using 4% articaine and epinephrin 1:100,000, De Morais *et al*. (2) also found no hemodynamic changes. The duration of articaine is surpassed only by long-acting anesthetics, such as bupivacaine, etidocaine, and ropivacaine, which have severe side effects for the central nervous system and cardiovascular system (20). No such effects were found in the present systematic review beyond tachycardia stemming from a possible vagal syncope. 

Side effects were more frequent with articaine (12 events) compared to lidocaine (seven events). With articaine, there were four cases of temporary paresthesia (20,24), seven cases of substantial trismus on the seventh day of the postoperative period (11,23), and one episode of tachycardia followed by hypotension and bradycardia with sudoresis (likely vagal syncope) of mild intensity and self-limited (22). The only side effect found with lidocaine was persistent trismus in seven patients. Despite these side effects, no complication was permanent and no cases required additional treatment. The resolution was spontaneous in all cases and there was no statistically significant difference in the number of events between the two drugs.

A large part of the studies had some concerns with regards to the risk of bias (11,18-22,25,26) and although all studies were randomized clinical trials, few described the randomization method employed (10,22,25). The majority (10,18,20-22,24,25,27) performed blinding, but none described the method used. Despite not detailing the blinding method, the evaluation of these studies was not negatively affected according to the analysis based on the RoB 2, as there was no deviation from the intended interventions. Two studies (23,28) that did not have blinding were classified as having a high risk of bias for the criterion, as the absence of blinding could exert a negative impact on the analysis of the outcomes.

The choice of a drug should be based on both the benefits and risks. Success, intraoperative pain, duration of the anesthetics, and postoperative pain are considered important to the choice of a local anesthetic and articaine proved superior to lidocaine regarding these aspects, with a high degree of reliability. Although more side effects were found with articaine, which is a critical outcome, the difference in comparison to lidocaine was not statistically significant.

To assist the scientific community and healthcare providers, future clinical trials should be conducted comparing articaine to other drugs, such as mepivacaine, which, according to the literature, also has good anesthetic potency. Another important point is that several of the studies included in the present review (18,19,21,25,27) did not comment on the occurrence of complications. As any drug can cause side effects, it is essential for clinical trials to provide this information in order to orient the use or not of the drug, while also taking into account any comorbidities found in the patients. Moreover, a more detailed description of the methods employed should be performed in clinical trials, as the articles were quite flawed with regards to the detailing of the randomization and blinding processes, which are very important criteria for this type of study.

The choice of a local anesthetic should not be performed only by analyzing its qualities; its side effects should also be taken into consideration so that the choice can be based on both the benefits and risks. In the present review, articaine is confirmed as an excellent option for blocking the inferior alveolar nerve during the removal of lower third molars based on its excellent clinical results. Moreover, it was found to be a safe drug.

Only two articles among the fourteen included in this review presented data on positioning of the lower third molar according to the Pell & Gregory and Winter classification, however, they do not correlate positioning factors with the outcomes addressed in this study (Boonsiriseth *et al*., 2017; Bhagat *et al*., 2014). Therefore, this data was not included in this review.

The present systematic review demonstrated that articaine is superior to lidocaine for use in lower third molar surgeries due to its higher success rate, shorter onset, greater control of intraoperative pain, and longer duration of the anesthetic effect. Although presenting more side effects than lidocaine, articaine did not cause any permanent or serious complication and the meta-analysis revealed no significant difference in the number of events.
